# Social Media Discussions About Robotic Total Knee Arthroplasty: Cross-Sectional Analysis

**DOI:** 10.2196/69883

**Published:** 2025-10-09

**Authors:** Charles Desgagné, Jordan J Levett, Lior M Elkaim, John Antoniou

**Affiliations:** 1 Orthopaedic Research Laboratory, Lady Davis Institute, McGill University, 845 Rue Sherbrooke O, Montreal, QC, H3A 0G4, Canada, 1 (514) 398-4455; 2 Faculty of Medicine, McGill University, Montreal, QC, Canada; 3 Faculty of Medicine, University of Montreal, Montreal, QC, Canada

**Keywords:** social media, twitter, knee replacement, arthroplasty, robotic, robotic surgery, patient, clinician, researcher

## Abstract

**Background:**

The advent of robotic total knee arthroplasty (TKA) in the field of orthopedics has caused much discussion on social media. As social media grows, its platforms are becoming an increasingly popular medium for health care–related discussions.

**Objective:**

This study aimed to better understand the current public discussion about robotic TKA on social media. We aimed to characterize these discussions by analyzing their contributors, the general sentiment, the temporal trends, and the content.

**Methods:**

A comprehensive search of the Twitter database for academic research was performed from inception (March 2006) to April 1, 2023, to identify all tweets related to robotic TKA. General data regarding the tweets and the accounts were retrieved. ChatGPT-4o (OpenAI) was used to categorize the post’s content and the accounts into different categories developed via iterative testing. The content was categorized using a rule-based classification algorithm developed using Python to assign categories based on keyword presence, phrase matching, and syntactic patterns. Regarding the accounts, an automated keyword-based rule engine was implemented in Python to classify accounts based on the account’s name and description. We used a lexicon-based natural language processing Python library, via ChatGPT-4o, to assign a sentiment to the tweets and conducted subgroup sentiment analysis.

**Results:**

A total of 2000 tweets were retrieved for analysis. Account analysis revealed that the most prevalent account categories were “medical professionals” (619/2000, 31.0%), “patients and community” (274/2000, 13.7%), and “media and publications” (268/2000, 13.4%). Content analysis revealed that the most prevalent tweet themes were “technology and innovation” (550/2000, 27.5%), “advertising and promotion” (176/2000, 8.8%), and “research and data” (172/2000, 8.6%). Sentiment analysis showed that 61.6% (1231/2000) of the tweets had a positive sentiment, while 9.2% (183/2000) were neutral, and 29.3% (586/2000) had a negative sentiment. Accounts categorized as “institutions” had the highest prevalence of positive sentiment (165/229, 72.1%), while accounts categorized as “media and publications” had the highest prevalence of negative sentiment (88/268, 32.8%). The number of tweets relating to robotic TKA has been steadily rising since 2016, with a peak incidence of 402 (20.1%) tweets published in 2022.

**Conclusions:**

The increased number of tweets with a positive sentiment suggests a positive outlook toward robotic TKA. Institutions had the highest prevalence of positive sentiment, suggesting a possible bias toward positive reporting of robotic TKA, likely for commercial reasons. Media and publications had the highest prevalence of negative sentiment, which may represent skepticism and bias toward negative reporting on robotic technologies in health care. Medical professionals contributed significantly to the discussion about robotic TKA, while patient involvement was relatively small. The number of tweets relating to robotic TKA has been steadily growing since 2016, which indicates that robotic TKA has been gaining in popularity over recent years.

## Introduction

### Background

Total knee arthroplasty (TKA) is one of the most common procedures in orthopedics [1]. As of 2019, more than 480,000 primary TKAs were being performed under Medicare in the United States alone [2]. TKA involves removing the affected articular surfaces of the knee and replacing them with polyethylene, metal, or other prosthetic components [3]. TKA is known to be a safe procedure with a high satisfaction rate [4]; however, up to 20% of patients remain unsatisfied with their clinical result [5]. Several factors can contribute to patient dissatisfaction, such as instability of the prosthesis, medial joint laxity, and persisting pain, among others [4]. A multitude of solutions has been proposed to improve patient satisfaction rates. One of the most promising solutions has been the use of robotic technology. Robotic technology has already been used to improve surgical outcomes in various fields such as general surgery, obstetrics and gynecology, cardiology, and ophthalmology [6]. Over recent years, robotic-assisted TKA has gained traction as an interesting avenue to improve surgical outcomes and patient satisfaction by aiding in precisely realigning the mechanical axis, enhancing ligament balance, and optimizing implant positioning [7].

### Rationale

The advent of robotic TKA in the field of orthopedics has caused much discussion on social media. As social media grows, its platforms are becoming an increasingly popular medium for health care–related discussions. A study published in 2020 found that 17.1% of the health information proposed by Google in response to arthroplasty questions originated from social media websites [8]. Among all social media platforms, Twitter (subsequently rebranded as X) is the social media platform that is most used for health care communication [9]. Authors included in this study have previously characterized, via analysis of sentiment, content, contributors, and temporal trends, social media discussions regarding glioblastoma, cervical myelopathy, anterior cruciate ligament injury prevention, epilepsy, pediatric spine surgery, among others [10-14]. Previous research has also analyzed social media discussions regarding robotic total hip arthroplasty [15], but no previous study has analyzed social media discussions surrounding TKA or robotic TKA, despite TKA being one of the most common procedures in orthopedics [1]. By analyzing the content of these discussions, it is possible to reveal important trends, such as decreased trust in a specific procedure, which can subsequently be addressed by physicians, researchers, and the industry. This study aimed to better understand the current public discussion about robotic TKA on social media. Similar to previously published literature, we aimed to characterize these discussions by analyzing their sentiment, content, contributors, and temporal trends.

## Methods

### Search Strategy

The methodology used in this study is based on previous studies detailing social media discourse in various health care fields [11,13,16]. A thorough search of Twitter’s application programming interface (API) for academic research was performed from inception (March 2006) to April 1, 2023. We used the following search terms: (total knee replacement OR total knee arthroplasty OR knee replacement OR knee arthroplasty OR TKA OR TKR) AND (robot OR robotic).

### Accounts

We excluded duplicate accounts, accounts with fewer than 10 tweets, bots, and accounts with fewer than 15 followers from our study. User accounts were classified as bots either based on usernames explicitly indicating their bot nature or if all tweets from the account consisted of retweets with identical formatting. The following data were extracted for further analysis: account location, number of followers, number of tweets, and year joined Twitter. We used ChatGPT-4o [17] to categorize the accounts into different categories. The following prompt was inserted into ChatGPT-4o:

*I am in the process of writing a scientific article looking at Tweets discussing robotic total knee arthroplasty on Twitter. The excel file included in this message contains the raw data of 2000 tweets. Row B contains the account username, while row E contains the description of the author’s account. Classify the accounts into the following categories, used by previous studies: “foundation,” “medical center,” “business,” “journal,” “MD/researcher,” “news channel,” “patient/caregiver,” “support group” and “other.” If there are better categories that can be used, please use them and compare the data. Tell me how I can make this prompt better for you.*

The output stated that the categories detailed in Table S1 in Multimedia Appendix 1 significantly decreased the number of accounts categorized as “other,” and ensured clarity, mutual exclusivity, and comprehensive coverage of account categories. Chat-GPT4o used an automated keyword-based rule engine that was implemented in Python to classify accounts based on the account’s name and description. The “other” category included accounts that either had no available descriptions and whose names could not hint toward their origin, or accounts that could not be categorized in any of the proposed categories. A sample of 20 accounts was reviewed by one independent evaluator (CD) for the validity of the classification.

**Table 1.** Examples of tweets categorized as positive, neutral, and negative.

Sentiments	Example of tweet content
Positive	*“Our patients travel great distances to consult with our orthopedic team... approved to purchase VELYS robotic-assisted solution.”* *“Dr. X (censored) has successfully conducted robotic hip & knee joint replacement surgery on three patients.”*
Neutral	*“Orthopaedic surgeons are now performing total knee replacement surgeries using robotic systems that do not require CT scans.”* *“M. X (censored) sharing her experience after total knee replacement surgery by Dr. X (censored).”*
Negative	*“Completely agree — present cost of the robot is not justified. But if studies show it improves outcome, cost may come down like electric cars. Otherwise, it will be remembered as a very expensive marketing tool.”* *“Nothing will replace human touch, but what can this robot do that the human hand has failed to achieve?”*

### Tweets

After duplicate removal, all retrieved tweets were considered eligible. For each tweet, the date of the tweet, the number of retweets, replies, quotes, and likes, as well as the presence of media, tagging, hashtags, and links, were retrieved. All included tweets were then categorized by ChatGPT-4o into one of the content categories presented in Table S2 in Multimedia Appendix 1. The following prompt was inserted into Chat-GPT4o:

*“Using the same raw data as previously used, categorize the content (row G) into one of the following categories, used by previous studies: “advertising,” “awareness,” “experience,” “research,” and “other.” If there are better categories that can be used, please use them and compare the data. Tell me how I can make this prompt better for you.*

The output stated that the categories detailed in Table S2 in Multimedia Appendix 1 significantly decreased the number of accounts categorized as “other,” and ensured clarity, mutual exclusivity, and comprehensive coverage of tweet content. A rule-based classification algorithm was developed by Chat-GPT4o using Python to assign categories based on keyword presence, phrase matching, and syntactic patterns. A sample of 20 tweets was reviewed by one independent evaluator (CD) for validity of the classification.

### Statistical Analysis

We used descriptive statistics (median, IQR) to analyze the different data points retrieved regarding the accounts and the tweets. Statistical significance was reached if *P*<.05. R (R Core Team; version 4.1.3) was used to conduct all statistical analyses.

### Sentiment

ChatGPT-4o was used to determine the tone of the tweet (ie, the sentiment). The following prompt was inserted into ChatGPT-4o:

*Using the same raw data as previously used, please classify the text of the tweet (row G) into the following sentiment categories: negative, neutral or positive. Please detail the methodology used to analyze the sentiment of the Tweets*.

ChatGPT-4o subsequently analyzed the tone of the tweets using TextBlob, a lexicon-based natural language processing Python library [18] that uses a predetermined dictionary of words, which analyzes data semantically. Chat-GPT4o opted to use the same lexicon-based natural language processing library (TextBlob) as used by previous studies with similar methodologies [11,12,14]. Regarding the sentiment analysis, TextBlob attributed a score to the tweet’s polarity and subjectivity. The polarity score was used to represent the sentiment. A polarity score of −1 represented the most negative tweets, whereas a score of +1 represented the most positive tweets. The subjectivity score (range 0‐1) was used to assess whether a tweet was objective (score of 0) or subjective (score of 1). Finally, the algorithm assigned a label based on the polarity scores; a score <0 represented a negative sentiment, a score of 0 represented neutral sentiment, and a score >0 represented a positive sentiment. Subjectivity scores were retained to provide additional context for future qualitative analysis but were not used as primary classification criteria. Once the sentiment analysis was completed, we conducted subgroup analyses within the different account categories to identify trends. Table 1 presents examples of tweets categorized as positive, neutral, and negative.

### Ethical Considerations

The data obtained in our study was freely obtained from Twitter’s API for Academic Research. As this is publicly available data, this study does not meet the inclusion criteria for approval by an institutional board of the Canadian Tri-Council Policy Statement [19]. All social media identifiers (names, handles) were removed to preserve confidentiality.

## Results

### Overview

A total of 7339 tweets were obtained using our search criteria. A random sample of 2000 tweets was retrieved for further analysis. Regarding the accounts from which the tweets originated, the median number of followers was 858 (IQR 207-3276) and the median number of tweets per author was 3129.5 (IQR 863.5-11581). Regarding the tweets, the mean retweet count was 0.80 (SD 2.95), the mean reply count was 0.27 (SD 1.45), the mean like count was 3.01 (SD 10.50), and the mean quote count was 0.10 (SD 0.51).

### Accounts

Manual review of account categorization revealed a concordance of 80% (16 tweets out of 20). Account analysis revealed a high prevalence of accounts categorized as “medical professionals” (619/2000, 31%). Despite our efforts to develop a categorization scheme that covered most of the accounts, the “other” category remained prevalent (523/2000, 26.2% of total accounts). Accounts categorized as “other” included accounts with descriptions such as the following: “*I live in San Jose, ca.*” The name of the account has been censored for privacy purposes, but does not provide information regarding account origin. Such accounts are difficult to categorize in a succinct categorization scheme. Finally, accounts related to “institutions,” “media and publications,” and “patients and community” were similarly prevalent at around 13% (229-274/2000) each. These findings are summarized in Table 2.

**Table 2.** Percentage of accounts per account category (N=2000).

Account categories	Accounts, n (%)
Industry and business	87 (4.4)
Institutions	229 (11.5)
Media and publications	268 (13.4)
Patients and community	274 (13.7)
Other	523 (26.2)
Medical professionals	619 (31.0)

### Tweets

Manual review of tweet categorization revealed a concordance of 75% (15 tweets out of 20). Content analysis revealed a high prevalence of tweets categorized as “other” (676/2000, 33.8%), while “technology and innovation” accounted for 27.5% (550/2000). “Advertising and promotion,” “research and data,” and “experience” accounted for about 8% (148-176/2000) of the content, respectively, while “news and institutional highlights,” “awareness, education and events,” and “surgical practice and technique” were much less prevalent. These findings are summarized in Table 3.

**Table 3.** Percentage of tweets per content category.

Content categories	Percentage of posts
Surgical practice and technique	3.4
Awareness, education, and events	5.3
News and institutional highlights	5.4
Experience	7.4
Research and data	8.6
Advertising and promotion	8.8
Technology and innovation	27.5
Other	33.8

### Sentiment

On sentiment analysis, 183 of 2000 (9.2%) tweets had a neutral sentiment, 586 of 2000 (29.3%) tweets had a negative sentiment, and 1231 of 2000 (61.6%) tweets had a positive sentiment. Subgroup analysis was notable for a high prevalence of positive sentiment within the “institution” group (165/229, 72.1%). Meanwhile, “industry and business” had the highest prevalence of neutral sentiment (12/87, 13.8%), and the “media and publications” group had the highest prevalence of negative sentiment (88/268, 32.8%). These findings are summarized in Table 4.

**Table 4.** Subgroup sentiment analysis.

Author category	Negative, n/n (%)	Neutral, n/n (%)	Positive, n/n (%)
Industry and business	23/87 (26.4)	12/87 (13.8)	52/87 (59.8)
Institutions	51/229 (22.3)	13/229 (5.7)	165/229 (72.1)
Media and publications	88/268 (32.8)	21/268 (7.8)	159/268 (59.3)
Medical professionals	199/619 (32.1)	41/619 (6.6)	379/619 (61.2)
Other	152/523 (29.1)	71/523 (13.6)	300/523 (57.4)
Patients and community	78/274 (28.5)	23/274 (8.4)	173/274 (63.1)

### Temporal Dynamics

The number of tweets relating to robotic TKA has been steadily rising over the years, with a peak incidence of 402 of 2000 (20.1%) tweets published in 2022. Table 5 presents the number of Tweets for each year from 2010 to 2023. The number of tweets for the year 2023 relates to the number of tweets published before April 1, 2023, when the data extraction was done.

**Table 5.** Number of tweets per year from 2010 to 2023 (N=2000).

Year	Tweets, n (%)	
2010‐2013	108 (5.4)	
2014	30 (1.5)	
2015	63 (3.2)	
2016	55 (2.8)	
2017	142 (7.1)	
2018	314 (15.7)	
2019	155 (7.8)	
2020	249 (12.5)	
2021	341 (17.1)	
2022	402 (20.1)	
2023	141 (7.1)	

## Discussion

### Principal Findings

This is the first study to explore the landscape of social media discussions regarding robotic TKA. Our data demonstrated a high prevalence of tweets with a positive sentiment (1231/2000, 61.6%). Accounts categorized as “institutions” had the greatest prevalence of positive sentiment (165/229, 72.1%), while accounts categorized as “media and publications” had the highest prevalence of negative sentiment (88/268, 32.8%). Medical professionals contributed significantly to the discussion about robotic TKA (619/2000, 31.0% of accounts), while patient involvement was relatively small (274/2000, 13.7% of accounts). The number of tweets relating to robotic TKA has been steadily growing since 2016.

### Sentiment

Our sentiment analysis showed that 61.6% (1231/2000) of the tweets had a positive sentiment, while 9.2% (183/2000) were neutral, and 29.3% (586/2000) had a negative sentiment. Studies with similar methodologies have consistently reported lower percentages of tweets with a positive sentiment. For instance, similar studies assessing tweets regarding pediatric spine surgery, cervical myelopathy, and glioblastoma reported a positive sentiment in 46.5%, 38.6%, and 43.6% of the tweets, respectively [10,11,14]. A recent study about social media discussions of anterior cruciate ligament injury prevention revealed a positive sentiment in 45.1% of the tweets [12]. TKAs are known to have a high satisfaction rate [4], and the elevated prevalence of positive sentiment in the tweets discussing robotic TKA on Twitter could be a reflection of this high satisfaction rate. Furthermore, subgroup analysis revealed that “medical professionals” had a highly prevalent positive sentiment within their tweets (379/619, 61.2%), and they represented the most prevalent account category (619/2000, 31.0% of the accounts).

On subgroup analysis, our data showed that “institutions” had the highest prevalence of positive sentiment (165/229, 72.1%). Indeed, tweets from institutions often boasted about new robotic-assisted TKA capacities and were likely biased towards positive phrasing for commercial reasons. Tweets with a positive sentiment and originating from an account categorized as “institution” included the following:

*great news at X (censored institution) yesterday dr X (censored) performed first navio robotic assisted total knee replacement.*

Previous literature has reported that hospital promotions tend to overstate benefits, and sometimes omit risks associated with robotic surgery [20,21]. On the other hand, the group with the highest prevalence of negative sentiment within their tweets was “media and publications,” which likely represents skepticism from news outlets toward robotic technologies in surgery. Tweets with a negative sentiment and originating from an account categorized as “media and publications” included the following:

*knee surgery isnt as bad as it used be […] now that robots are giving docs helping hand*.

Although this might initially appear as a positive tweet, the phrasing presents knee surgery as a previously “bad” operation, which brings the natural language processing algorithm to correctly assign it a negative tone. Previous literature has reported similar trends from news outlets. For instance, a paper published in the *Journal of Robotic Surgery* reported that, out of 27 journal articles analyzed, all 27 reported disadvantages of robotic surgery, while only 7/27 (26%) reported findings favoring robotic approaches [22].

### Concordance Analysis

Discordance between manual categorization and ChatGPT-4o’s categorization of the content and the accounts appears to be related to insufficient context and syntax errors. For instance, the following account description was categorized as “other” by ChatGPT-4o:

*‘’In compliance with federal and state privacy laws, do not include any private health information in any message you send us through this channel*.

This account was related to a medical institution and was thus manually categorized as “institution” by the independent reviewer. However, because of missing context and keywords, ChatGPT-4o categorized it as “other.” Similarly, the following content was categorized as “other” by Chat-GPT4o:

*experienced high volume surgeons robotic total knee arthroplasty was longer more costly procedure than manual total knee arthroplasty with similar lengths stay complications.*

This tweet referred to a research paper and was thus manually categorized as “research and data” by the independent reviewer. However, considering the lack of keywords and syntax errors, ChatGPT-4o classified it as “other.”

### Actors Discussing Robotic Total Knee Arthroplasty

Our results showed that most of the accounts discussing robotic TKA on Twitter were related to the following categories: “medical professionals” (619/2000, 31%), “other” (523/2000, 26.2%), and “patients and community” (274/2000, 13.7%). Similar studies have reported widely different results regarding contributors to social media discussions. In a study with a similar methodology about pediatric spine surgery, it was found that 79% of the tweets originated from a patient or caregiver, while only 3.3% from a physician or researcher [14]. In another similar study about anterior cruciate ligament injury prevention on Twitter, patients and caregivers represented 55.7% of the accounts [12]. On the other hand, Elkaim et al [11] reported that, similarly to our data, 37.8% of the accounts discussing degenerative cervical myelopathy on social media originated from medical doctors or researchers, while 23.5% originated from patients or caregivers. The significant contribution of medical professionals to the discussion surrounding robotic TKA on Twitter may be explained by the fact that robotic-assisted surgery constitutes a highly debated topic within the health care field, notably regarding doubts about the cost-benefit ratio [23] and some evidence demonstrating nonsuperiority to laparoscopic surgery [24,25]. Regarding the decreased contribution of “patients and community” to the discussion, it is possible that patients and caregivers discussing robotic TKA on Twitter do not specify the robotic-assisted aspect of the surgery in their tweets and are subsequently not included in our search of the API. To verify this hypothesis, a future study could be designed to compare TKA and robotic TKA discussions on Twitter.

### Content of the Tweets

The diversity of themes observed in the tweets reflects the multifaceted nature of discussions surrounding robotic TKA on Twitter. The most prevalent tweet categories were “other” (676/2000, 33.8%), followed by “technology and innovation” (550/2000, 27.5%) and “advertising and promotion” (176/2000, 8.8%). Tweets categorized as “other” included tweets such as:

*why do they call it knee replacement not were turning you into robot” or “even robotic knee replacement needs your clever multimodal analgesic cocktail.*

Such content is difficult to categorize in a succinct categorization scheme. Other studies about social media discussions regarding different medical procedures have used different categorization schemes for content. Fortunately, a category dedicated to “research” is often found in other articles. Levett et al [14] found that 40.9% of tweets on Twitter relating to pediatric spine surgery discussed research, while Elkaim et al [11] found that 45% of tweets on Twitter relating to cervical myelopathy discussed research [11]. “Research and data” accounted for only 8.6% (172/2000) of the content related to robotic TKA. This might affect the overall accuracy of the content related to robotic TKA on social media, although most accounts discussing robotic TKA on Twitter were medical professionals, which might attenuate this lack of accuracy. As stated in the Methods section, many tweets could not be categorized into the categories proposed in our study because of their unclear general content.

### Temporal Dynamics

Our results showed that the number of tweets relating to robotic TKA increased substantially in the year 2020 (from 155 in 2019 to 249 in 2020) and continued to grow in the years that followed (up to 402 in 2022). Similar studies have reported different trends. Elkaim et al [11] reported a surge in tweet volume regarding degenerative cervical myelopathy in 2018‐2019 and a significant decrease in 2020‐2021. Similarly, Levett et al [12] reported a surge in tweet volume regarding anterior cruciate ligament injury prevention in 2018‐2019 and a subsequent decrease in the following years. It appears that tweets relating to robotic TKA have also experienced a surge in 2018. However, the tweet count has been steadily rising since. It has already been reported in the literature that the presence of physicians has steadily grown on Twitter from the year 2016 to 2020 [26], and considering that “medical professionals” accounted for more than 30% (619/2000) of the accounts discussing robotic TKA on social media, it is unsurprising that robotic TKA has experienced increased popularity during this timeframe.

### Limitations and Directions for Future Research

While our study provides valuable insights into the discussions surrounding robotic TKA on social media, it is essential to address certain limitations. First, our results are based on a single social media platform. Although Twitter is considered the most popular social media platform for health care discussions [9], including more social media platforms would provide a more complete dataset. Including more social media platforms remains difficult, as Twitter is the only social media platform with easily accessible, extensive data. Second, a significant proportion of the accounts (523/2000, 26.2%) and the content (676/2000, 33.8%) were categorized as “other” because they were not compatible with the categories proposed by our study, or because they simply could not be classified (eg, no author description and a name unrelated to an account category). More studies are needed to establish standardized categorization schemes of tweets and accounts that can be reproduced in different health care domains. More research is also needed to compare different orthopedic procedures. This could reveal important trends, such as a decreased trust in a procedure, which can subsequently be addressed by physicians, researchers, and the industry.

### Conclusions

This is the first study to explore the landscape of social media discussions regarding robotic TKA. The increased number of tweets (61.6%) with a positive sentiment reflects a positive outlook towards robotic TKA on social media. Accounts categorized as “institutions” had the greatest prevalence of positive sentiment (165/229, 72.1%), suggesting a possible bias towards positive reporting of robotic TKA, likely for commercial reasons. Accounts categorized as “media and publications” had the highest prevalence of negative sentiment (88/268, 32.8%), which may represents a skepticism and a bias toward negative reporting on robotic technologies in health care. Medical professionals contributed significantly to the discussion about robotic TKA (619/2000, 31.0% of accounts), while patient involvement was relatively small (274/2000, 13.7% of accounts). The number of tweets relating to robotic TKA has been steadily growing since 2016, which indicates that robotic TKA has been gaining in popularity over recent years.

## Supplementary material

10.2196/69883 Multimedia Appendix 1 Description of account and content categories.

## Abbreviations

API: application programming interface
TKA: total knee arthroplasty
