# Supplementary material for: Social Media Discussions About Robotic Total Knee Arthroplasty: Cross-Sectional Analysis
Source: JMIR Infodemiology. 2025 Oct 9;5:e69883. doi: 10.2196/69883 (PMC12510438; doi:10.2196/69883)
Supplement: Multimedia Appendix 1 [file infodemiology-v5-e69883-s001.docx]

**Table S1.** Description of the account categories.

| **Categories** | **Includes** | **Keywords** |
| --- | --- | --- |
| Medical professionals | MDs^a^, surgeons, fellows, researchers, scientists | “surgeon,” “doctor,” “MD,”^a^ “orthopaedic” “physician” |
| Institutions | Hospitals, medical centers, clinics, universities, academic departments | “hospital,” “university,” “institute,” “center,” “clinic” |
| Industry and business | Health tech companies, startups, marketers, consultants, private clinics | “company,” “robotics,” “solutions,” “tech” |
| Media and publications | Journals, news outlets, magazines, medical media | “media,” “journal,” “news,” “press” |
| Patients and community | Patients, caregivers, support groups, advocates, foundations | “patient,” “advocate,” “recovery,” “support” |
| Other | All remaining unclassifiable accounts | N/A |

**Table S2.** Description of the content categories.

| **Categories** | **Definition** | **Keywords** |
| --- | --- | --- |
| Advertising and promotion | Tweets aimed at promoting a service, clinic, product, or practitioner. Includes appointment calls-to-action or marketing language. | “Book now,” “Call us,” “Offering robotic surgery,” “Visit our clinic” |
| Experience | Personal accounts from patients or surgeons describing procedures, recovery, outcomes, or emotions. | “I underwent,” “My story,” “Treated with,” “Pain-free,” “I get to...” |
| Research and data | References to studies, trials, publications, or data-driven claims about robotic TKA^a^. Often shared by professionals or journals. | “RCT,^b^” “Study,” “Published,” “Evidence-based,” “Trial results” |
| Technology and innovation | Discussions of surgical systems, robotics, new devices, AI^c^, or FDA-approved technologies related to robotic TKA. | “Surgical robot,” “FDA^d^ cleared,” “Guidance system,” “AI in surgery” |
| Awareness, education and events | Educational tweets, webinars, explainer threads, awareness campaigns, conference or event participation and invitations. | “Join our webinar,” “Awareness day,” “Educational video,” “Symposium” |
| News and institutional highlights | Institutional or professional achievements, press releases, milestones, or announcements from hospitals, universities, or medical groups. | “First to offer,” “Hospital milestone,” “Press release,” “Featured in” |
| Surgical practice and technique | Tweets detailing surgical methods, implant positioning, workflows, planning tools, or intraoperative decisions and philosophies. | “Precision cut,” “Workflow,” “Planning software,” “Implant technique” |
| Other | Tweets that do not clearly match the above categories, including vague commentary, unrelated content, or incomplete context. | “Interesting perspective,” “This is wild,” unclear or general content |
